# Immunopathology of Fatal Human Variegated Squirrel Bornavirus 1 Encephalitis, Germany, 2011–2013

**DOI:** 10.3201/eid2506.181082

**Published:** 2019-06

**Authors:** Dennis Tappe, Jonas Schmidt-Chanasit, Jessica Rauch, Petra Allartz, Christiane Herden

**Affiliations:** Bernhard Nocht Institute for Tropical Medicine, Hamburg, Germany (D. Tappe, J. Schmidt-Chanasit, J. Rauch, P. Allartz);; German Centre for Infection Research, Hamburg (J. Schmidt-Chanasit);; University of Giessen, Giessen, Germany (C. Herden)

**Keywords:** Bornavirus, VSBV-1, variegated squirrel bornavirus 1, encephalitis, caspase 3, apoptosis, cytotoxic T cells, p53, immunopathology, Germany, viruses, zoonoses

## Abstract

Variegated squirrel bornavirus 1 (VSBV-1) is a zoonotic virus that causes fatal encephalitis in humans who are infected after contact with exotic squirrels. We analyzed the brain lesions and the immune responses in all 4 known human cases that showed panencephalitis. Inflammatory infiltrates in areas positive for VSBV-1 RNA and antigen consisted of CD4+ and CD8+ T cells, with perivascular B-cell accumulation. Strong microglial response and bizarre astroglial expansion were present. Areas of malacia contained neutrophils and foamy microglia and macrophages. Immunopathologic examination during infection showed cleavage of caspase 3 in brain cells adjacent to CD8+ cells and widespread p53 expression, hallmarks of apoptosis. Cerebrospinal fluid analyses over time demonstrated increasing protein concentrations and cell counts, paralleled by pathologic lactate elevations in all patients. The most severe cerebrospinal fluid and histologic changes occurred in the patient with the highest viral load, shortest duration of disease, and most medical preconditions.

Variegated squirrel bornavirus (VSBV-1; family *Bornaviridae*, species *Mammalian 2 orthobornavirus*) is a new zoonotic virus that causes severe and eventually fatal encephalitis in humans. VSBV-1 was discovered retrospectively in 2015 in a cluster of 3 fatal encephalitis cases among private breeders of exotic variegated squirrels (*Sciurus variegatoides*) in eastern Germany (*1*). Phylogenetic analyses showed that this virus forms a separate lineage within the *Bornaviridae* family. VSBV-1 is related to, but distinct from, the classical Borna disease virus 1 (BoDV-1; species *Mammalian 1 orthobornavirus*). Recently, also in retrospect, VSBV-1 was shown to be responsible for fatal limbic encephalitis in a zoo animal caretaker after contact with an exotic Prevost’s squirrel (*Callosciurus prevostii*) in northern Germany (*2*). The virus is of unknown origin (*3*) and most likely transmitted by bites and scratches of infected exotic squirrels of the subfamilies *Sciurinae* from Central America and *Callosciurinae* from Southeast Asia (*1*,*2*) in holdings in Europe. The animals are asymptomatic and show high viral RNA loads, not only in the brain but also in organs capable of secretion and excretion, such as the kidney, urinary bladder, skin, and oropharynx (*1*,*3*,*4*). The distribution of viral RNA and antigen in the human brain has been described only in 1 patient (the patient with limbic encephalitis) (*2*). The pathophysiology of human VSBV-1 infection and the immune response toward the virus in humans is unknown.

We here summarize clinical data of all 4 known human VSBV-1 encephalitis cases and describe the distribution of VSBV-1 in different brain areas as determined by real-time reverse transcription PCR (RT-PCR) and immunohistochemical (IHC) analysis in the initial encephalitis cluster. We focus on the characterization of the central nervous system (CNS) immunologic response to VSBV-1 by IHC analyses of immune cells in the brain of all patients, as well as by examination of cerebrospinal fluid (CSF) reactions over time during the disease.

## Patients, Materials, and Methods

### Encephalitis Cases

Three patients were male private breeders (62–72 years of age) of exotic squirrels (*1*), and 1 patient was a female zoo animal caretaker (45 years of age) who had occupational contact with exotic squirrels (*2*). All 4 patients had had subacute, slow-onset, progressive, and eventually fatal encephalitis. Duration of their illnesses ranged from 2 to 4 months. In all 4 patients, fever or chills, initial abdominal symptoms, and later respiratory problems occurred, in addition to signs of CNS involvement, such as confusion and psychomotor slowing. Myoclonus occurred in 2 patients and ataxia in 3 patients. Illnesses progressed to coma and death. Changes in the brain or meninges were visible by magnetic resonance imaging late in the course of the disease, >4 weeks after onset of the first symptoms (Table 1).

**Table 1:** Characteristics of patients with fatal variegated squirrel bornavirus 1 encephalitis, Germany

Characteristic	Patient 1	Patient 2	Patient 3	Patient 4
Year of illness (reference)	2011 (*1*)	2013 (*1*)	2013 (*1*)	2013 (*2*)
Age, y/sex	63/M	62/M	72/M	45/F
Duration of illness, mo.	3	2	4	3
Medical preconditions	Hypertension	Hypertension, type 2 diabetes, renal insufficiency	Hypertension, obesity	None
Predominant symptoms	Myoclonus, tetraparesis, coma	Myoclonus, ataxia, coma	Ocular paresis, ataxia, coma	Ataxia, coma
Geographic area (state) of infection	East Germany (Saxony-Anhalt)	East Germany (Saxony-Anhalt)	East Germany (Saxony-Anhalt)	North Germany (Schleswig-Holstein)
Squirrel contact	Private breeder of variegated squirrels	Private breeder of variegated squirrels	Private breeder of variegated squirrels	Zoo animal caretaker, contact with a Prevost’s squirrel

From all 4 patients, formalin-fixed paraffin-embedded (FFPE) brain tissue was available; from patient 1, only a brain biopsy sample was available because no autopsy was granted. However, FFPE blocks from internal organs of patients 2–4 (myocardium, lungs, liver, kidney, spleen, bone marrow, intestine) were also available for analyses. Ethics clearance was obtained from the local ethics board (Medical Board of Hamburg, no. PV5616).

### Molecular Assays

VSBV-1–specific quantitative real-time RT-PCR was performed from FFPE tissues as previously described (*1*,*2*). Tissue samples from different brain areas from patients 1–3 and samples of internal organs from patients 2 and 3 were analyzed in this study. For patient 4, real-time RT-PCR analyses for VSBV-1 RNA in different brain areas and internal organs had been performed previously (*2*).

### Histologic and IHC Analyses

Standard hematoxylin and eosin staining was performed from FFPE CNS tissues of all 4 patients and from FFPE internal organs of patients 2–4. IHC for VSBV-1 antigen was performed for patients 1–3 on different CNS tissue samples and for patients 2–4 on internal organ samples. For patient 4, IHC for VSBV-1 only from brain has been performed previously (*2*). For VSBV-1 IHC, polyclonal rabbit antiserum against viral N and P proteins was used (*2*).

Further IHC studies were performed for all 4 patients in CNS tissue samples to demonstrate glial fibrillary acidic protein (GFAP, 1:100; Zytomed Systems, https://www.zytomed-systems.de), CD3 (1:400; Epitomics Abcam, https://www.abcam.com), CD20 (1:150; Agilent, https://www.agilent.com), CD4 (1:30; Cell Marque, http://www.cellmarque.com), CD8 (1:20; Cell Marque), CD68 (1:100; Agilent), CD177 (1:33; Zytomed Systems), HLA-DR (1:50; DakoAgilent, https://www.agilent.com/en/dako-products), inducible nitric oxide synthase (iNOS, 1:100; Zytomed Systems), cleaved caspase-3 (CC3, 1:300; Cell Signaling Technology, https://www.cellsignal.de), TdT-mediated dUTP-biotin nick end labeling (TUNEL assay, 1:10; SigmaAldrich/Merck, https://www.sigmaaldrich.com), granzyme B (1:50; Agilent), Ki67 (1:20; DakoAgilent), and p53 (1:50; DakoAgilent). After pretreatment with buffers containing Trilogy (medac diagnostika, http://www.medac-diagnostika.de) at 95°C for CD177, EDTA (pH 8 for CD4 and p53), or citrate (pH 6 for CD3, CD20, CD8, CD68, HLA-DR, iNOS, TUNEL, and Ki67) and endogenous peroxidase blocking, the FFPE tissue sections were incubated with the respective antibodies in Antibody Diluent Solution (Zytomed) at 4°C overnight. This step was followed by incubation with the DCS-AEC 2 Component Detection Kit and 3-amino-9-ethylcarbazole substrate (DCS, http://www.dcs-diagnostics.de) for immunoperoxidase staining or the DCS AP Detection Kit and Fast Blue substrate (DCS) for immunophosphatase staining. Brain tissue from 5 patients without encephalitis served as negative controls and human lymphatic tissue as a positive control for immunologic staining of immune cells.

### CSF Analyses

Total CSF protein and lactate concentrations and cell counts were performed following standard procedures from all 4 patients over time while the patients were still alive. Patients were seen in different hospitals with an initially unknown disease and CSF analyses were performed in different time intervals. Intrathecal antibody synthesis was demonstrated by testing for oligoclonal bands (isoelectric focusing and immunofixation of IgG parallel in serum and CSF) and by calculation of the IgM, IgA, and IgG index (ratio between CSF and serum immunoglobulin class after correcting for albumin concentrations in the CSF and serum).

## Results

### Real-time RT-PCR Detection of VSBV-1 RNA

Real-time RT-PCR for VSBV-1 RNA was positive in different brain areas in all patients but negative in all internal organs. The highest viral loads were detected in midbrain regions, hippocampus, and temporal cerebral cortex (Table 2).

**Table 2:** Real-time RT-PCR results for variegated squirrel bornavirus 1 RNA in different brain areas and internal organs, Germany*

Sample	Cycle threshold		
	Patient 1	Patient 2	Patient 3
Cortical biopsy	31.2	30.8	NA
Cortex frontal	NA	31.1	29.9
Cortex temporal	NA	27.9	28.9
Striatum	NA	28.9	29.7
Hippocampus	NA	28.3	29.4
Midbrain/substantia nigra	NA	26.4	29.4
Cerebellum	NA	34.6	29.9
Olfactory bulb	NA	NA	29.7
Myocardium	NA	Neg	Neg
Lungs	NA	Neg	Neg
Kidney	NA	Neg	Neg
Liver	NA	Neg	Neg
Spleen	NA	Neg	Neg

*All materials tested were formalin-fixed paraffin-embedded tissue specimens. Not all tissues were available from all patients because no autopsy was performed on patient 1 and no biopsy sample was taken from patient 3. From patient 4, real-time RT-PCR analyses for variegated squirrel bornavirus 1 RNA in different brain areas and internal organs were performed earlier (*2*). NA, not available; neg, negative; RT-PCR, reverse transcription PCR.

### Histologic and IHC Analyses of Brain Tissue and Internal Organs in VSBV-1 Infection

**Figure 1:**
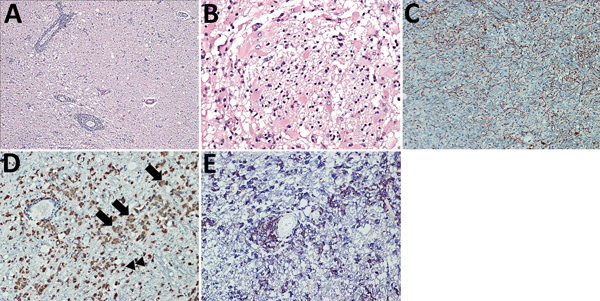
Brain inflammation in patients with fatal variegated squirrel bornavirus 1 encephalitis. A) Brain section showing mononuclear cell infiltration and tissue edema. Hematoxylin and eosin stain; original magnification ×100. B) Depiction of glial cell activation in a brain biopsy sample of malacia. Astrocytes appear bizarre and enlarged. Hematoxylin and eosin stain; original magnification ×400. C) Demonstration of glial fibrillary acidic protein (GFAP). Immunoperoxidase stain with hematoxylin counterstain; original magnification ×200. D) Predominantly foamy cells (lipid-laden microglia or macrophages [arrows]) and a few bushy microglia (arrowheads) as shown by marked CD68 positivity throughout the inflamed regions. Immunoperoxidase stain with hematoxylin counterstain; original magnification ×200. E) HLA-DR (brown) and CD68 positivity (blue) indicating many reactive microglia and macrophages. Immunoperoxidase and immunophosphatase stains; original magnification ×200.

In general, tissue analyses showed the histologic picture of a panencephalitis. Most commonly, the temporal and insular lobes and the basal ganglia and midbrain were affected. Areas of tissue necrosis (malacia), edema, perivascular lymphocyte cuffing, parenchymal lymphocyte infiltration, and glial activation were found in the brains of all patients in the white and gray matter in a random distribution (Figure 1, panel A). Astrocytes appeared bizarre and enlarged (Figure 1, panel B). The most severe changes were found in the brain tissue of patient 2. Furthermore, satellitosis, occasional neuronophagia, single-cell necrosis of Purkinje cells (patients 2 and 4), loss of hippocampal pyramidal cell layer (patient 2), focally extensive hemorrhage (patient 2), and lipofuscin deposition were found. Histopathologic analyses of the internal organs in the patients showed no inflammatory or degenerative changes (data not shown). GFAP reactivity (Figure 1, panel C) confirmed the strong astroglial activation consisting of variably enlarged protoplasmic astrocytes which could be binucleated or multinucleated up to a bizarre appearance. Compared with negative control brains, patient brain tissues showed strong HLA-DR and CD68+ reactivity throughout the inflamed regions in predominantly foamy cells, which represent either lipid-laden microglia or macrophages, and in a few bushy microglia (Figure 1, panels D, E). In contrast, little iNOS production was seen (Appendix Figure 1).

**Figure 2:**
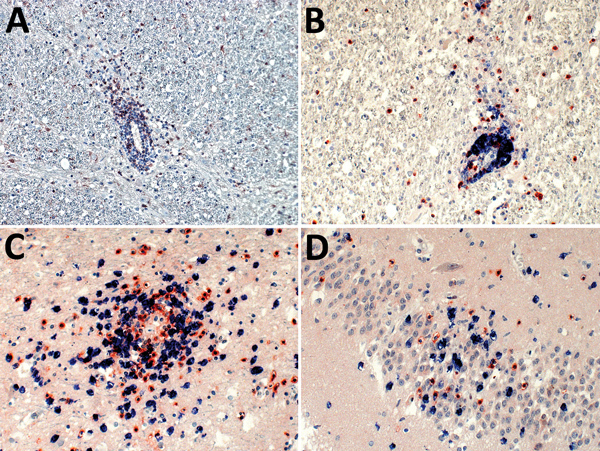
Demonstration of neutrophils, B cells, and CD4+ and CD8+ T-lymphocyte infiltration of the brain parenchyma during variegated squirrel bornavirus 1 infection. A) Demonstration of CD177+ neutrophils infiltrating the brain parenchyma from a blood vessel. Neutrophils are also seen scattered throughout the tissue during the infection. Immunoperoxidase stain with hematoxylin counterstain; original magnification ×200. B) Depiction of B cells (CD20, blue) and T cells (CD3, red) around a blood vessel. B cells showed mainly perivascular cuffing, and only a few were found in the brain parenchyma, whereas T cells were also seen in high numbers infiltrating the brain parenchyma. Immunoperoxidase and immunophosphatase stains; original magnification ×200. C, D) Mixed CD4+ (red) and CD8+ (blue) T-cell infiltrates around a blood vessel and invading adjacent tissue (C), and the hippocampus (D). Immunoperoxidase and immunophosphatase stains; original magnification ×400.

Invasion by neutrophils showed infiltration of the brain parenchyma from blood vessels in areas of malacia (Figure 2, panel A); these CD177+ cells were also scattered throughout the brain tissue in such regions. CD20+ B cells were mainly seen as perivascular cuffing, and only a few were found in the tissue otherwise (Figure 2, panel B). In contrast, CD3+ T cells also occurred in high numbers in the brain parenchyma. The T-cell infiltrates consisted of CD4+ helper cells and CD8+ cytotoxic cells; neither type predominated (Figure 2, panels C and D). In control brain tissues, CD4+ cells were sparse in the parenchyma, but no CD8+ cells or granulocytes were present (data not shown).

**Figure 3:**
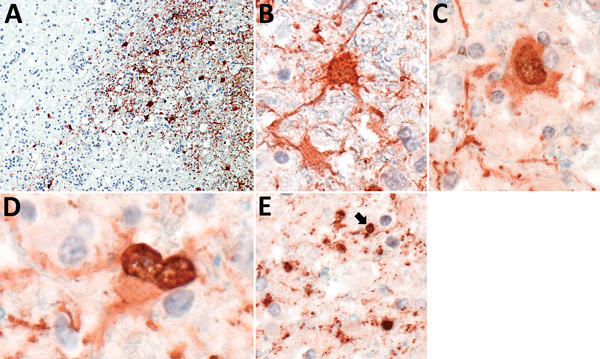
Presence of variegated squirrel bornavirus 1 (VSBV-1) antigen in neurons, astrocytes, and oligodendrocytes. A) Widespread presence of VSBV-1 antigen in brain tissue. Endothelial cells show no viral antigen. Immunoperoxidase stain with hematoxylin counterstain; original magnification ×200. B, C) Demonstration of VSBV-1 antigen in neurons. Immunoperoxidase stain with hematoxylin counterstain; original magnification ×600. D) VSBV-1 antigen in an astrocyte. Immunoperoxidase stain with hematoxylin counterstain; original magnification ×600. E) VSBV-1 antigen in an oligodendrocyte (arrow). Immunoperoxidase stain with hematoxylin counterstain; original magnification ×600.

In all patients, IHC analysis for VSBV-1 antigen showed areas of widespread neuropil, cytoplasmic, and nuclear staining (Figure 3, panel A). Antigen was found in neurons and astrocytes (Figure 3, panels B–D), mainly with enlarged appearance, and oligodendrocytes (Figure 3, panel E). Endothelial cells were always spared. IHC analysis detected VSBV-1 antigen in the brain areas that were also positive for VSBV-1 RNA. No positive VSBV-1 immunostaining was detected in the internal organs (data not shown).

**Figure 4:**
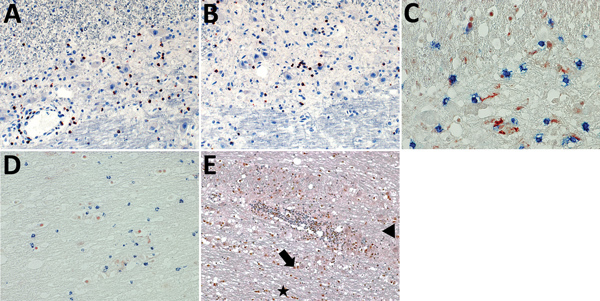
Induction of apoptosis in brain cells through association with granzyme B+/CD8+ cells, formation of cleaved caspase 3 and p53 up-regulation. A, B) Granzyme B+ cells in association with neurons and astrocytes during variegated squirrel bornavirus 1 infection. Immunoperoxidase stain with hematoxylin counterstain; original magnification ×400. C) Demonstration of brain cell apoptosis by shrinkage and positivity for cleaved caspase 3 (red) in close contact with CD8+ T cells (blue). Immunoperoxidase and immunophosphatase stains; original magnification ×600. D) Apoptotic cells positive for cleaved caspase 3 (red) in spatial association with granzyme B+ cytotoxic cells (blue). Immunoperoxidase and immunophosphatase stains; original magnification ×400. E) Widespread positivity for the p53 protein. Marked staining of perivascular mononuclear inflammatory cells, neurons (arrow), astrocytes (arrowhead), and rod cells (star) is shown. Immunoperoxidase stain with hematoxylin counterstain; original magnification ×200.

Staining for granzyme B showed immune cells adjacent to large neurons and glia cells (Figure 4, panels A, B) during VSBV-1 infection. Widespread apoptosis was shown by positive CC3 staining and shrinkage of brain cells in close association with CD8+ T lymphocytes and granzyme B positivity (Figure 4, panels C, D), as well as positive TUNEL assays (not shown). In addition, widespread p53 positivity was seen mainly in perivascular mononuclear inflammatory cells, some neurons, astrocytes, and rod cells throughout the affected areas (Figure 4, panel E) but only sparse Ki67 positivity in perivascular inflammatory mononuclear cells (Appendix Figure 2). In control brain tissues, no CC3, TUNEL, p53, or Ki67 positivity was seen (data not shown).

### CSF Changes During VSBV-1 Infection

**Figure 5:**
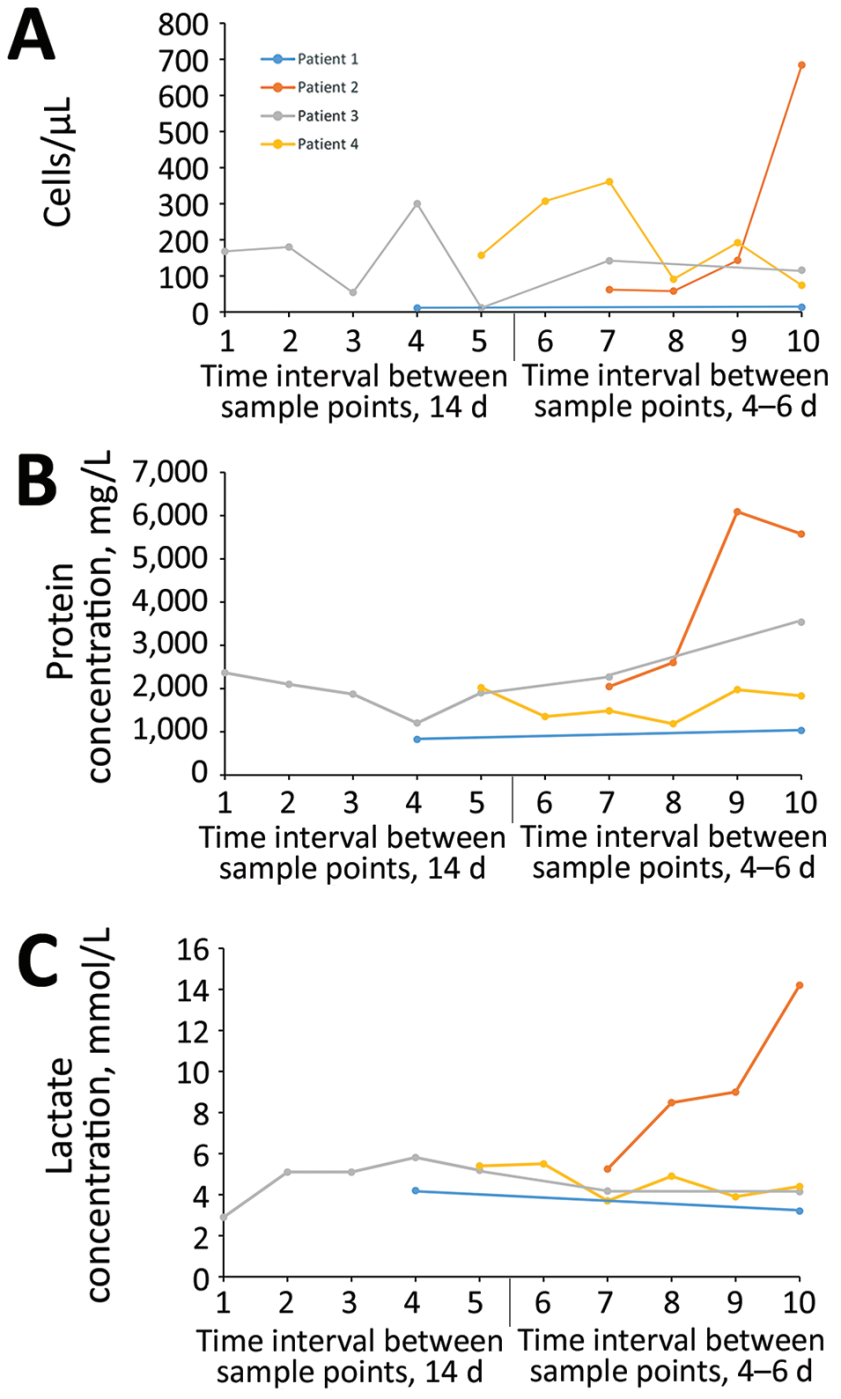
Cerebrospinal fluid (CSF) parameter changes over time during human infection with variegated squirrel bornavirus 1 (VSBV-1) encephalitis. CSF analyses were performed at different time intervals (every 14 days or every 4–6 days) because the patients were seen in different hospitals. Patients had different durations of the disease (2–4 months), including a nonspecific prodromal phase during which no lumbar puncture was performed. A) Changes in CSF cell counts during the course of disease. An undulating lymphocytic pleocytosis is seen in patients 3 and 4, whereas in patient 1 only a mild cell count elevation is visible. In contrast, patient 2 shows a very prominent cell increase. B) Constantly increasing CSF protein levels during infection. A marked increase is demonstrated for patient 2. C) Lactate concentration changes in the CSF. Although pathologic levels decreased slowly over time in patients 1, 3, and 4, levels in patient 2 increased prominently.

Standard clinical CSF analyses of the patients over time showed lymphocytic pleocytosis (range 11–361 cells/μL, median 142 cells/μL [reference <5/μL]) (Figure 5, panel A), markedly elevated protein concentrations (range 824–6,092 mg/L, median 1,976 mg/L [reference <500 mg/L]) (Figure 5, panel B), lactate level elevation (range 2.9–8.47 mmol/L, median 5.1 mmol/L [reference <2.1 mmol/L]) (Figure 5, panel C), and blood–brain barrier dysfunction. All patients showed increasing CSF protein concentrations over time. Patients 1, 3, and 4 demonstrated slow decreases of pathologic lactate concentrations during the observation period. In contrast, patient 2 exhibited a drastic increase of lactate levels and CSF cell counts. In patient 1, cell counts were only very mildly elevated; in patients 3 and 4, cell counts changed in an undulating manner over time. In all patients, increased intrathecal concentrations of IgM, IgA, and IgG were found, with positive IgM and IgG indices and oligoclonal IgG bands that demonstrated intrathecal antibody production.

## Discussion

In this study, we analyzed VSBV-1 distribution in the brains of human patients. We focused on the local immune response in brain tissue, as well as CSF reactions during infection. We broadly expanded previously published scarce general histologic descriptions of fatal human VSBV-1 infection and initial CSF laboratory findings.

In patients 1–3, we found VSBV-1 RNA in all brain regions that were available: cerebral cortex, basal ganglia, hippocampus, midbrain, cerebellum, and olfactory bulb. In patient 4, VSBV-1 RNA has been shown in the limbic system, basal ganglia, and cerebellum, but not in the cerebral cortex (*2*). The highest viral loads were found in midbrain regions, hippocampus, and temporal cortex, whereas the lowest viral loads were seen in the cerebellum. These findings are in line with previous findings for natural and experimental infections of animals with BoDV-1 (*5*,*6*). Viral loads in our study were determined by quantitative real-time RT-PCR from FFPE sections known to have higher cycle thresholds than native samples because of the formalin fixation. In addition to tissue, CSF has been shown to be RT-PCR positive in patients from whom CSF had been stored (patients 3 [*1*] and 4 [*2*]). In concordance with previous results of patients 1 (*1*) (biopsy only) and 4 (*2*) (different brain regions), we showed that viral nucleic acid could be demonstrated in every brain area that was also positive for viral antigen by IHC in all patients. This finding also fits with the simultaneous detection in animals experimentally or naturally infected with BoDV-1. Patients with cerebral cortex infection as shown by RT-PCR and IHC analysis had myoclonus (patients 1 and 2), and those with cerebellar and basal ganglia infection showed ataxia (patients 2–4). In none of the patients was viral RNA or antigen detected in the internal organs. This finding does not exclude an infection of other organs that could not be analyzed in our study. However, experimental infection of immunocompetent rats is also strictly neurotropic and did not result in any peripheral organ spread of BoDV-1 (*7*,*8*). 

For unknown reasons, all patients had initial abdominal and later respiratory signs or symptoms. Involvement of the peripheral nervous system, which we did not examine because of lack of preserved material, could be possible. The only cranial nerve available for analysis, the olfactory bulb of patient 3, was positive for viral RNA and antigen. Whether the infection of the olfactory bulb implies a potential intranasal entry route or resulted from centrifugal spread of VSBV-1 from the brain remains unclear.

The histologic features of VSBV-1 infection of the brain in the 4 patients consisted of multifocal malacia, edema, inflammatory perivascular, and parenchymal cell infiltration, as well as strong microglial and astroglial activation. The enlarged astrocytes seen in our study resemble the bizarre appearance of astrocytes in JC virus–induced progressive multifocal leukencephalopathy cases where they tend to undergo apoptosis and might be misdiagnosed as tumor cells (*9*). The inflammatory infiltrates in the brain parenchyma of the VSBV-1 encephalitis patients mainly consisted of CD4+ and CD8+ T cells, accompanied by neutrophils and macrophages, and a few B cells. The pronounced infiltration by CD8+ T cells and the close association of CD8+ T cells with neuronal cells and granzyme B+ cells with astrocytes are signs of a prominent cytotoxic response of the human immune system to the VSBV-1 infection. Orthobornaviruses cause nonlytic infections of CNS cells; clinical disease and histopathologic lesions in animals after infection with BoDV-1 are caused by a T-cell–mediated immunopathology and not by the viral replication itself (*10*–*12*). The presence of CD4+ and CD8+ T cells were already demonstrated in horses naturally infected with BoDV-1 (*12*) and in experimentally infected rats and mice (*8*,*13*–*16*). In horses and rats, CD4+ cells dominated the perivascular inflammatory infiltrates, and CD8+ T cells were found in parenchymal infiltrates. In our study of human VSBV-1 infections, we did not observe this effect; rather, CD4+ and CD8+ cells were equally distributed around blood vessels and in the brain parenchyma. Such effects, however, might be due to different time points of histologic examination as horses and experimental animals are euthanized early in the course of disease. Similar to findings for equine BoDV-1 infections (*15*), a predominance of CD3+ cells and rarity of neutrophils and B cells in the parenchymal infiltrates was seen in the human VSBV-1 infections.

The hypothesis of a T-cell–mediated immunopathogenesis causing clinical signs and symptoms during human VSBV-1 infection is highlighted by the detection of CC3- and TUNEL- positive cells as apoptotic markers in close association with the infiltrating cytotoxic immune cells. Caspase 3 is a member of the cysteine protease family and, in its activated cleaved form, CC3, a key mediator of neuronal apoptosis. CC3 is affected by both the cell surface receptor-mediated and the mitochondrial apoptosis pathways and thus plays an important role in the final common pathway of apoptosis (*17*,*18*). In neonatal rats infected with BoDV-1, hippocampal caspase 3 activation has been observed (*19*), and neuronal apoptosis has been shown in the neocortex, hippocampus, and cerebellum (*20*). However, these animals were immune incompetent at the time of infection. Caspase 3 inhibitors were experimentally neuroprotective in rodents during cerebral ischemia and necrosis (*18*,*21*) but are not licensed as medical therapeutics. Caspase inhibitors repressed apoptosis in a BoDV-1–infected nonneuronal cell line but could not block viral transcription (*22*). For experimental BoDV-1 infections in rodents, infected neurons were not lysed by cytotoxic T cells (*6*,*23*). Thus, further investigation is needed on whether the immunopathologic mechanisms during human VSBV-1 infection differ from those observed in animal BoDV-1 infection or might instead result from a potential proinflammatory state of the CNS. A longer duration of inflammation and clinical disease in human infections also could be a reason. The observed p53 up-regulation in our study seems to be associated with the inflammatory process, but whether this represents an enhancement of the aforementioned apoptotic processes or is a beneficial event remains to be elucidated. For BoDV-1, the viral phosphoprotein can interfere with p53 indirectly through host HMBG1 and it was therefore suggested that BoDV-1 inhibits p53 activity (*24*). Viral interference with the p53 system in the CNS is also seen in JC virus infections (*25*); however, the proliferation marker Ki67 that is overexpressed in progressive multifocal leukencephalopathy (*26*) was only sparsely demonstrated in our study.

The CSF changes in the patients in our study (lymphocytic pleocytosis, protein elevation, blood–brain barrier dysfunction, and local antibody production) are typical for a viral infection of the CNS. The lactate concentration elevations in the CSF of all patients are most likely reflecting the severe inflammation and necrosis. Especially in patient 2, the person with the most severe histologic changes (inflammation and presence of viral antigen) and the shortest duration of disease (2 months vs. a 3- to 4-month course of disease in the other patients), the prominent increases of lactate levels, protein concentrations, and cell counts that were seen over time may serve as a marker for a rapid VSBV-1 disease progression. This patient also had the most medical preconditions and the highest viral load in central brain areas. In the other patients, the protein concentration elevations were paralleled by a rather moderate pleocytosis. All patients showed markedly elevated CSF protein levels that further increased over time and intrathecal antibody production. In patient 4, the specificity of such CSF IgG antibodies for VSBV-1 was demonstrated recently (*2*).

In conclusion, our results strongly demonstrate a severe inflammatory response pointing toward a T-cell–mediated immunopathology in human VSBV-1 infection. This process involves immune cells also seen in BoDV-1–associated lesions in natural and experimental infection, accompanied by evidence of brain cell apoptosis. A rapid clinical course in 1 patient could be linked to higher viral load and more severe inflammation.

Appendix Additional images from patients with fatal variegated squirrel bornavirus 1 encephalitis, Germany, 2011–2013.
