# Supplementary material for: Immunopathology of Fatal Human Variegated Squirrel Bornavirus 1 Encephalitis, Germany, 2011–2013
Source: Emerg Infect Dis. 2019 Jun;25(6):1058–65. doi: 10.3201/eid2506.181082 (PMC6537742; doi:10.3201/eid2506.181082)
Supplement: Appendix — Additional images from patients with fatal variegated squirrel bornavirus 1 encephalitis, Germany, 2011–2013. [file 18-1082-Techapp-s1.pdf]

Article DOI: https://doi.org/10.3201/eid2506.181082

# Immunopathology of Fatal Human Variegated Squirrel Bornavirus 1 Encephalitis, Germany, 2011–2013

## Appendix

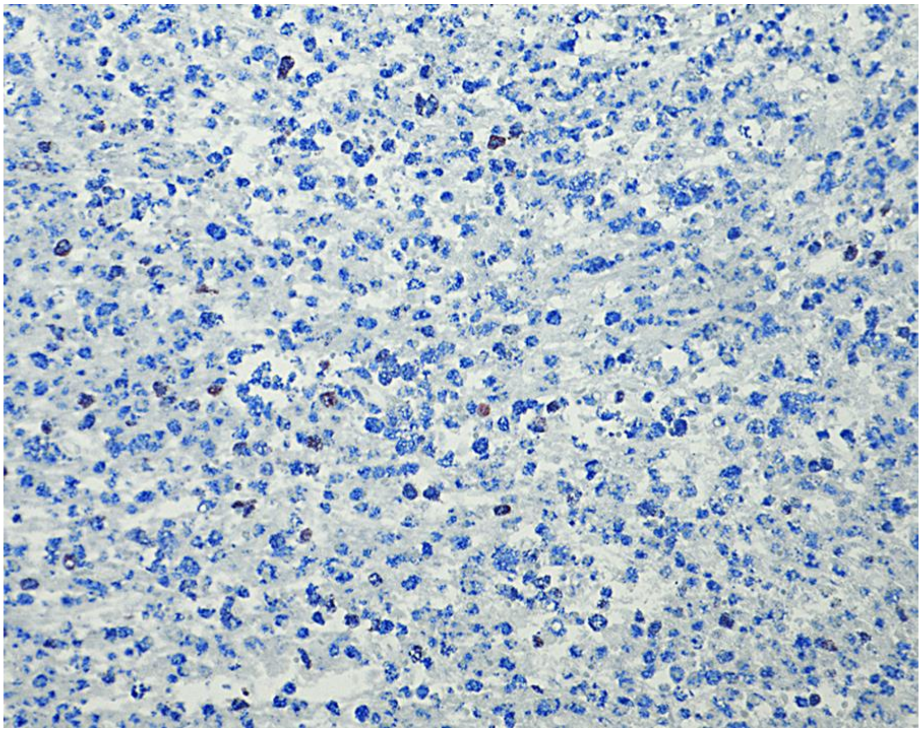

**Appendix Figure 1.** Immunohistochemical demonstration of inducible nitric oxide synthase (iNOS). Only little iNOS production is found. Immunoperoxidase stain with hematoxylin counterstain. Original magnification ×400.

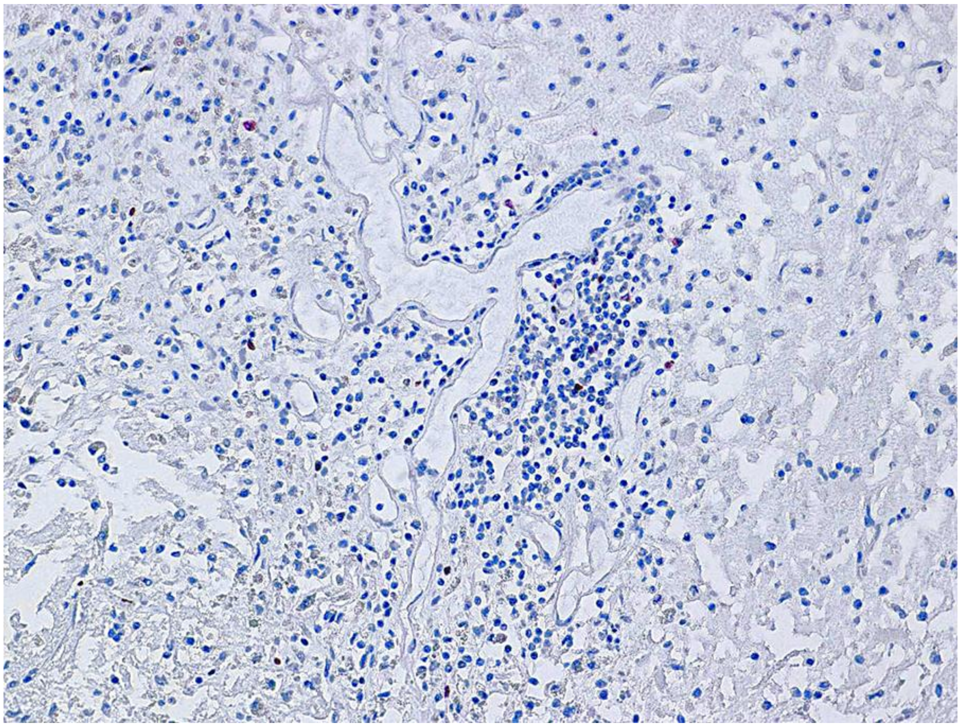

**Appendix Figure 2.** Immunohistochemical demonstration of the proliferation marker Ki67. A sparse positivity for the proliferation marker Ki67 is seen. Perivascular inflammatory mononuclear cells positive for Ki67 were few and only found in proximity to blood vessels. Immunoperoxidase stain with hematoxylin counterstain. Original magnification ×200.
